# Veterans with Gulf War Illness exhibit distinct respiratory patterns during maximal cardiopulmonary exercise

**DOI:** 10.1371/journal.pone.0224833

**Published:** 2019-11-12

**Authors:** Jacob B. Lindheimer, Dane B. Cook, Jacquelyn C. Klein-Adams, Wei Qian, Helene Z. Hill, Gudrun Lange, Duncan S. Ndirangu, Glenn R. Wylie, Michael J. Falvo

**Affiliations:** 1 War Related Illness and Injury Study Center, Department of Veterans Affairs New Jersey Health Care System, East Orange, New Jersey, United States of America; 2 William S. Middleton Memorial Veterans Hospital, Madison, Wisconsin, United States of America; 3 Department of Kinesiology, University of Wisconsin-Madison, Madison, Wisconsin, United States of America; 4 New Jersey Medical School, Rutgers Biomedical and Health Sciences, Newark, New Jersey, United States of America; 5 Department of Neurology, Mount Sinai Beth Israel, New York, New York, Unites States of America; 6 Kessler Foundation, West Orange, New Jersey, United States of America; Teesside University/Qatar Metabolic Institute, UNITED KINGDOM

## Abstract

**Introduction:**

The components of minute ventilation, respiratory frequency and tidal volume, appear differentially regulated and thereby afford unique insight into the ventilatory response to exercise. However, respiratory frequency and tidal volume are infrequently reported, and have not previously been considered among military veterans with Gulf War Illness. Our purpose was to evaluate respiratory frequency and tidal volume in response to a maximal cardiopulmonary exercise test in individuals with and without Gulf War Illness.

**Materials and methods:**

20 cases with Gulf War Illness and 14 controls participated in this study and performed maximal cardiopulmonary exercise test on a cycle ergometer. Ventilatory variables (minute ventilation, respiratory frequency and tidal volume) were obtained and normalized to peak exercise capacity. Using mixed-design analysis of variance models, with group and time as factors, we analyzed exercise ventilatory patterns for the entire sample and for 11 subjects from each group matched for race, age, sex, and height.

**Results:**

Despite similar minute ventilation (*p* = 0.57, η^2^_p_ = 0.01), tidal volume was greater (*p* = 0.02, η^2^_p_ = 0.16) and respiratory frequency was lower (*p* = 0.004, η^2^_p_ = 0.24) in Veterans with Gulf War Illness than controls. The findings for respiratory frequency remained significant in the matched subgroup (*p* = 0.004, η^2^_p_ = 0.35).

**Conclusion:**

In our sample, veterans with Gulf War Illness adopt a unique exercise ventilatory pattern characterized by reduced respiratory frequency, despite similar ventilation relative to controls. Although the mechanism(s) by which this pattern is achieved remains unresolved, our findings suggest that the components of ventilation should be considered when evaluating clinical conditions with unexplained exertional symptoms.

## Introduction

Increased minute ventilation (${\dot{V}}_{\text{E}}$, l·min^-1^) secondary to physiological, pathophysiological or psychological stimuli is well-recognized. Less understood, however, are the independent changes in tidal volume (V_T_) and respiratory frequency (*f*_R_) that comprise ${\dot{V}}_{\text{E}}$; variables that are often overlooked [1]. As reviewed by Tipton and colleagues [1], a small but growing number of studies suggest that that V_T_ and *f*_R_ are under differential regulation in response to stressors, such as exercise, with each contributing uniquely to exercise hyperpnoea. For example, Nicolò and colleagues [2] recently demonstrated that *f*_R_, unlike V_T_, responds rapidly to changes in workload during high-intensity cycling and recovery, is independent from metabolic factors ($\dot{V}{\text{O}}_{2}$ and $\dot{V}{\text{CO}}_{2}$), and is strongly associated with perceived exertion. These findings support earlier work [3–6] suggesting that V_T_ is determined more by metabolic factors than *f*_R_. Understanding the differential control of V_T_ and *f*_R_ is of clinical relevance [1], and may provide unique insight for the interpretation of cardiopulmonary exercise testing (CPET).

A primary indication for CPET is the evaluation of undiagnosed exercise intolerance, particularly when symptoms are disproportionate with cardiopulmonary function [7]. Such a scenario has been described in military veterans with Gulf War Illness (GWI)–a chronic multisymptom illness affecting 25–32% of veterans deployed to Operations Desert Storm and Shield in 1990–1991 [8]. Although the etiology of GWI is incompletely understood, deployment related exposures (e.g., pesticides, nerve agents, oil well fires) are suspected to contribute [8]. As such, population-based studies indicate significantly greater respiratory symptoms between deployed and non-deployed Gulf War veterans [9] but similar pulmonary function [10]. Despite a clear indication for performing CPET (i.e., symptoms disproportionate to function), to our knowledge only two studies have utilized CPET for diagnostic purposes in GWI [11, 12], only one of which provided detailed CPET results [12, 13]. Numerous other studies have employed maximal or submaximal exercise testing in veterans with GWI as a stressor [13–21] to elucidate underlying mechanisms of GWI. These studies have provided considerable insight into the pathophysiology of GWI, but ventilatory patterns during CPET were beyond their purview and thus not reported. Therefore, our understanding of ventilatory patterns (${\dot{V}}_{\text{E}}$, V_T_ and *f*_R_) during exercise in GWI is nascent.

Cook and colleagues have previously found that veterans with GWI report enhanced pain sensitivity in response to exercise [16] and rate exercise as more effortful [13] relative to controls. Given that *f*_R_ is strongly associated with perceived exertion and provides a valid index of physical effort in healthy adults [2–4], we hypothesized that veterans with GWI may have an exaggerated *f*_R_ response to exercise that has not previously been recognized. An excessive increase in *f*_R_, and therefore ${\dot{V}}_{\text{E}}$, is an energetically inefficient strategy that may contribute to exercise-induced symptoms reported in this population. In this context, the present study was designed to compare maximal CPET performance between veterans with GWI and controls with respect to exercise ventilatory patterns (${\dot{V}}_{\text{E}}$, V_T_, and *f*_R_). Should between-group differences be detected as hypothesized, these findings may provide support for pursuing therapeutic strategies (i.e., breathing techniques) to ameliorate exertional symptoms and enhance exercise tolerance in veterans with GWI.

## Materials and methods

### Participants

Thirty-four individuals volunteered to participate in this study, including 20 cases of GWI (GWI+) and 14 controls (GWI-). GWI case status was determined using the Centers for Disease Control (CDC) and Kansas criteria [22]. In brief, cases must endorse moderate-to-severe symptoms in ≥ 3 domain areas (i.e., fatigue, pain, neurological/cognitive/mood, skin, gastrointestinal and respiratory) that began after 1990 and persisted for ≥ 1 year. Comorbid conditions (e.g., diabetes, heart disease, stroke, etc.) that may account for chronic symptoms were excluded per case definition [23], and were also excluded for control participants. In addition to meeting CDC and Kansas criteria, GWI+ cases were also required to be clinically fatigued as determined by the Fatigue Severity Scale [24]. Controls (GWI-) included both deployed veterans (n = 4) and civilians (n = 10). All participants completed a medical history that included self-reported physical activity (minutes per week) and the Veterans version of the Short Form 36 Health Survey [25] for descriptive purposes. Experimental procedures described below were reviewed and approved by the Department of Veterans Affairs New Jersey Health Care System’s Institutional Review Board, and all participants provided informed written consent.

### Spirometry

Participants were asked to abstain from consuming meals or caffeine for at least 4 hours prior to arriving to our laboratory. Spirometry was performed in accordance with standard guidelines [26] using commercially available equipment (Cosmed Quark PFT; Rome, Italy) that was calibrated prior to each participant. Spirometric indices of forced vital capacity (FVC), forced expiratory volume in one second (FEV_1_), and FEV_1_/FVC were obtained and expressed as a percent of predicted [27].

### Cardiopulmonary exercise testing

Resting 12-lead electrocardiogram (ECG) (Cosmed T12x; Rome, Italy) and blood pressure via manual auscultation were acquired during quiet rest. CPET was then performed on a cycle ergometer (Ergoline Ergoselect 100; Germany) using a ramp protocol (15 watts·min^-1^) and pedal cadence of 50–70 rpms until volitional exhaustion [7]. Before, during and after CPET, all participants were asked to rate their perceived exertion (6–20) and breathlessness (0–10) using the Borg scales. Maximal effort was defined as meeting two or more of the following criteria: 1) ≥ 1.1 respiratory exchange ratio, 2) ≥ 85% age-predicted heart rate, 3) ≥ 17 perceived exertion, or 4) $\dot{V}{\text{O}}_{2}$ plateau or decline despite increasing workload.

Pulmonary gas-exchange ($\dot{V}{\text{O}}_{2}$ and *$\dot{V}{\text{CO}}_{2}$*), ${\dot{V}}_{\text{E}}$, V_T_, *f*_R_, and end-tidal CO_2_ (P_ET_CO_2_) were obtained breath-by-breath using an oro-nasal mask (V2 Series; Hans Rudolph, Shawnee, KS) with bidirectional turbine and metabolic cart (Cosmed Quark CPET; Rome, Italy) that was calibrated prior to each participant. Heart rate was obtained from 12-lead ECG and integrated with breath-by-breath data. The ventilatory anaerobic threshold (VAT) was determined using a modified V-slope method [28], and common CPET variables were obtained at peak exercise (30-sec time average) and reported by group. Our primary variables of interest (${\dot{V}}_{\text{E}}$, V_T_, and *f*_R_) were calculated at distinct periods relative to each individual’s peak exercise capacity ($\dot{V}{\text{O}}_{2}$ peak) to facilitate between-group comparisons across similar intensities (i.e., 0% (rest), 20%, 40%, 60%, 80%, and 100% $\dot{V}{\text{O}}_{2}$ peak). A 15-breath average, aligned to the time of the central breath of peak $\dot{V}{\text{O}}_{2}$, was used to compute the times of relative intensities. Analyses were performed using a custom-built program (MATLAB; Mathworks; Natick, MA).

### Statistical analysis

Participant characteristics, baseline pulmonary function and peak exercise variables were analyzed using independent *t*-tests for continuous variables and Fisher’s exact test for categorical variables. Normality assumptions were checked (Kolmogorov-Smirnov) for our primary dependent variables (${\dot{V}}_{\text{E}}$, V_T_, *f*_R_) across groups (GWI+ vs. GWI-) and time-points (0%, 20%, 40%, 60%, 80%, and 100% $\dot{V}{\text{O}}_{2}$ peak). We used separate mixed-design 2 (group) X 6 time ANOVA models to examine between-group differences for each dependent variable (α = 0.05). Degrees of freedom were adjusted (Greenhouse-Geisser) when the sphericity assumption was violated. Subsequent pairwise comparisons used a Bonferroni adjustment to control family-wise error. Effect sizes are expressed as partial eta squared (η^2^_p_) for ANOVA/ANCOVA models and Hedges’*d* for pairwise comparisons [29]. In addition, we also performed sub-group analysis using 11 subjects from each group to control for the main determinants of vital capacity—i.e., race, age, sex, and height. Demographics for matched pairs are provided in supporting information (S1 Table). Through matching, the average difference in vital capacity was within 200 mL. Analyses were performed using IBM SPSS Statistics (v. 25).

## Results

### Participant characteristics

Demographics, self-reported physical and mental health, spirometric indices, and resting heart rate, blood pressure, and arterial blood oxygen saturation are provided in Tables 1 and 2. Descriptive CPET information collected during exercise is provided in Tables 3 and 4. Data are presented as group means and standard deviations (mean ± SD).

**Table 1:** Comparison of mean (SD) demographics and self-reported physical and mental health between participants with positive and negative Gulf War Illness diagnoses.

	Full sample			Matched subgroup		
	Mean (SD)		Effect size (95% CI)	Mean (SD)		Effect size (95% CI)
	GWI + (n = 20)	GWI–(n = 14)		GWI + (n = 11)	GWI–(n = 11)	
**Age (years)**	50.4 (6.89)	52.14 (5.72)	-0.26 (-0.95, 0.42)	53.36 (7.74)	52.18 (5.83)	0.17 (-0.67, 1.01)
**Height (cm)**	173.17 (8.1)	168.14 (11.5)	-0.51 (-0.18., 1.21)	172.75 (9.32)	168.57 (10.42)	0.42 (-0.42, 1.27)
**Weight (kg)**	84.57 (14.7)	82.86 (14.1)	-0.12 (-0.57, 0.8)	80.34 (14.41)	85.83 (14.48)	-0.38 (-1.22, 0.46)
**Sex (male/female)**	17/3	9/5	n/a	8/3	8/3	n/a
**Body Mass Index (kg/m**^**2**^ **)**	28.08 (3.61)	29.51 (5.34)	-0.32 (-1.01, 0.37)	26.87 (3.88)	30.3 (5.12)	-0.76 (-1.62, 0.11)
**Physical Activity (min·wk**^**-1**^ **)**	102.75 (182.18)^a^	113.75 (122.61)	-0.07 (-0.78, 0.65)	60 (84.85)	105 (128.16)^a^	-0.42 (-1.31, 0.47)
**Smoking History (pack-years)**	7.51 (12.72)^b^	4.68 (7.83)	0.25 (-0.44, 0.95)	5.86 (12.61)^b^	5.14 (8.56)	0.07 (-0.79, 0.92)
**Fatigue Severity Score**	44.15 (15.72)	20.79 (12.41)	1.58 (0.8, 2.36)	38.18 (16.14)	19.18 (13.35)	1.28 (0.37, 2.2)
**Physical Composite Score**	41.26 (10.31)	55.24 (8.73)^b^	-1.4 (-2.18, -0.63)	42.95 (7.71)	54.56 (9.96)^b^	-1.31 (-2.26, -0.37)
**Kansas GWI Total Score**	34.35 (17.56)	5.15 (10.12)^b^	1.89 (1.05, 2.72)	28.64 (18.61)	6.30 (11.36)^b^	1.43 (0.47, 2.39)

**Table 2:** Comparison of mean (SD) spirometric indices, and resting heart rate, blood pressure, and arterial blood oxygen saturation between participants with positive and negative Gulf War Illness diagnoses.

	Full sample			Matched subgroup		
	Mean (SD)		Effect size (95% CI)	Mean (SD)		Effect size (95% CI)
	GWI + (n = 20)	GWI–(n = 14)		GWI + (n = 11)	GWI–(n = 11)	
**FEV**_**1**_ **(L)**	3.32 (0.82)^a^	3.14 (0.6)	0.25 (-0.45, 0.95)	3.03 (0.76)^b^	3.2 (0.6)	-0.24 (-1.1, 0.62)
**FEV**_**1**_ **(% predicted)**	96.5 (13.16)^a^	106.57 (15.96)	-0.68 (-1.4, 0.04)	95.8 (9.1)^b^	108 (12.26)	-1.08 (-2, -0.16)
**FVC (L)**	4.46 (0.95)^a^	3.93 (0.76)	-0.6 (-0.12, 1.31)	4.02 (0.84)^b^	4 (0.7)	0.03 (-0.83, 0.88)
**FVC (% predicted)**	101.83 (10.65)^a^	105.07 (13)	-0.27 (-0.97, 0.43)	100.1 (10.55)^b^	106.64 (10.11)	-0.61 (-1.49, 0.27)
**FEV**_**1**_ **/FVC**	0.75 (0.1)^a^	0.81 (0.1)	-0.77 (1.49, -0.04)	0.75 (0.1)^b^	0.81 (0.1)	-0.78 (-1.66, 0.11)
**FEV**_**1**_ **/FVC (% predicted)**	94.94 (11.61)^a^	101.79 (8.96)	-0.63 (-1.35, 0.08)	95.9 (10.04)^b^	101.73 (9.09)	-0.59 (-1.46, 0.29)
**Resting Heart rate (bpm)**	66 (12)	69 (12)	-0.25 (-0.93, 0.44)	63 (11)	71 (13)	-0.65 (-1.51, 0.21)
**Resting Systolic BP (mmHg)**	117 (10)	125 (13)	-0.71 (-1.41, 0)	115 (11)	128 (13)	-1.1 (-2, -0.21)
**Resting Diastolic BP (mmHg)**	73 (4)	77 (8)	-0.57 (-1.27, 0.13)	73 (5)	77 (9)	-0.58 (-1.43, 0.27)
**Resting SpO**_**2**_ **(%)**	97 (1)	98 (1)	-0.3 (-0.98, 0.39)	97 (2)	98 (1)	-0.13 (-0.97, 0.71)

**Note: BP** = blood pressure; **FEV**_**1**_ = forced expiratory volume in one second; **FEV**_**1**_ **/ FVC**: ratio of forced expiratory volume in one second to forced vital capacity; **FVC** = forced vital capacity; **GWI+** = positive diagnosis for Gulf War Illness; **GWI-** = negative diagnosis for Gulf War Illness; **n/a** = not applicable; **SpO**_**2**_: arterial blood oxygen saturation.

^a^Data missing for 2 participants

^b^Data missing for 1 participant

**Table 3:** Comparison of mean (SD) cardiopulmonary exercise data between participants with positive and negative Gulf War Illness diagnoses.

	Full sample			Matched subgroup		
	Mean (SD)		Effect size (95% CI)	Mean (SD)		Effect size (95% CI)
	GWI + (n = 20)	GWI–(n = 14)		GWI + (n = 11)	GWI–(n = 11)	
$\dot{V}{\text{O}}_{2\text{peak}}$ **(mL·min**^**-1**^ **)**	2017.77 (497.47)	1768.82 (553.62)	0.47 (-0.22, 1.16)	1904 (478.95)	1843.75 (582.38)	0.11 (-0.73, 0.95)
$\dot{V}{\text{O}}_{2\text{peak}}$ **(mL·kg·min**^**-1**^ **)**	24.13 (4.69)	21.28 (4.73)	0.59 (-0.1, 1.29)	24.13 (4.23)	21.34 (4.51)	0.62 (-0.24, 1.47)
**RER**_**peak**_	1.15 (0.11)	1.14 (0.09)	0.08 (-0.6, 0.76)	1.17 (0.12)	1.15 (0.1)	0.23 (-0.61, 1.07)
**Power**_**peak**_ **(watts)**	142.55 (32.33)	137.14 (40.51)	0.15 (-0.54, 0.83)	139.73 (32.16)	142.27 (42.97)	-0.06 (-0.9, 0.77)
**Heart rate**_**peak**_ **(bpm)**	152 (18)	143 (21)	0.44 (-0.25, 1.14)	148 (22)	142 (23)	0.24 (-0.59, 1.08)
**Heart rate**_**%max**_ **(%)**	87.42 (8.48)	83.51 (13.25)	0.36 (-0.33. 1.05)	86.74 (10.06)	83.34 (14.71)	0.26 (-0.58, 1.1)
**RPE**_**peak**_ **(0–20)**	15 (2)	16 (3)	-0.31 (-0.99, 0.38)	15 (3)	16 (3)	-0.16 (-1, 0.67)
**Dyspnea**_**peak**_ **(0–10)**	5 (2)	5 (2)	0.03 (-0.65, 0.71)	5 (3)	5 (2)	0.04 (-0.8, 0.87)

**Table 4:** Continued comparison of mean (SD) cardiopulmonary exercise data between participants with positive and negative Gulf War Illness diagnoses.

	Full sample			Matched subgroup		
	Mean (SD)		Effect size (95% CI)	Mean (SD)		Effect size (95% CI)
	GWI + (n = 20)	GWI–(n = 14)		GWI + (n = 11)	GWI–(n = 11)	
**Exercise duration (sec)**	588 (127)	564 (165)	0.16 (-0.52, 0.85)	574 (119)	578 (166)	-0.03 (-0.86, 0.81)
$\dot{V}{\text{O}}_{2}$ **at VAT (mL·min**^**-1**^ **)**	1077.2 (230.8)	1065.26 (259.6)^a^	0.05 (-0.67, 0.76)	1029.57 (267.37)^a^	1107.88 (283.98)	-0.27 (-1.16, 0.61)
$\dot{V}{\text{O}}_{2}$ **at VAT (mL·kg·min**^**-1**^ **)**	12.82 (2.33)	12.93 (1.74)^a^	-0.05 (-0.77, 0.66)	12.87 (2.87)^a^	12.86 (1.63)	0.004 (-0.88, 0.88)
**VAT (%** $\dot{V}{\text{O}}_{2\text{peak}}$ **)**	54.34 (7.9)	58.47 (8.77)^a^	-0.49 (-1.22, 0.24)	54.32 (6.56)^a^	56.34 (5.29)	-0.32 (-1.21, 0.56)
$\dot{\text{V}}\text{E}$ **at VAT (l·min**^**-1**^ **)**	27.26 (7.28)	28.89 (6.91)^a^	-0.22 (-0.94, 0.5)	25.11 (7.79)^a^	29.95 (7.23)	-0.62 (-1.52, 0.28)
**RER at VAT**	0.86 (0.11)	0.87 (0.06)^a^	-0.13 (-0.84, 0.59)	0.83 (0.12)^a^	0.88 (0.07)	-0.48 (-1.37, 0.42)
${\dot{V}}_{\text{E}}$ **/MVV (%)**	54.39 (9.97)	54.49 (14.87)	-0.01 (-0.69, 0.67)	57.29 (7.23)	55.32 (15.14)	0.17 (-0.72, 1.05)
**V**_**T**_ **/IC**	0.69 (0.13)	0.63 (0.14)	0.44 (-0.25, 1.13)	0.75 (0.10)	0.65 (0.16)	0.74 (-0.17, 1.65)

^a^Data missing for 2 participants

### Minute ventilation

#### Full sample

The ${\dot{V}}_{\text{E}}$ ANOVA model yielded a significant main effect for time (F_1.25,40.12_ = 236.63, *p* < 0.001, η^2^_p_ = 0.88). The group effect (F_1,32_ = 0.33, *p* = 0.57, η^2^_p_ = 0.01) and group-by-time interaction effect (F_1.25,40.12_ = 0.52, *p* = 0.51, η^2^_p_ = 0.02) were both non-significant.

#### Matched subgroup

The ${\dot{V}}_{\text{E}}$ ANOVA model yielded a significant main effect for time (F_1.23,24.57_ = 159.05, *p* < 0.001, η^2^_p_ = 0.89). The group effect (F_1,20_ = 0.66, *p* = 0.43, η^2^_p_ = 0.03) and group-by-time interaction effect (F_1.23,24.57_ = 0.33, *p* = 0.62, η^2^_p_ = 0.02) were both non-significant.

### Tidal volume

#### Full sample

**Fig 1:**
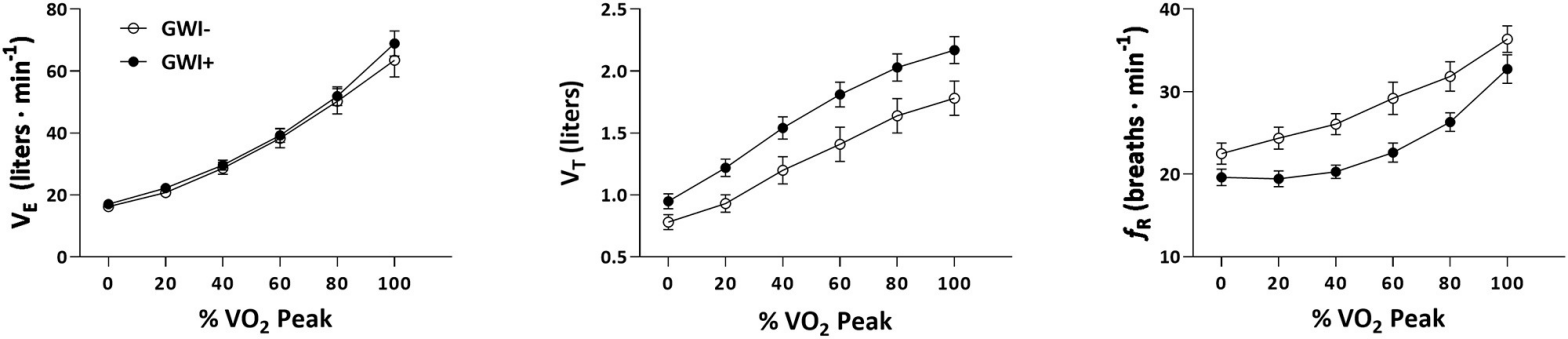
Time course of exercise ventilatory variables (${\dot{V}}_{\text{E}}$, V_T_, and *f*_R_) expressed as a function of relative intensity ($\dot{V}{\text{O}}_{2}$ peak) for the full sample (GWI+ = 20, GWI- = 14). Cases (GWI+) and controls (GWI-) are represented in filled and open circles, respectively. Values are mean ± SE.

The V_T_ ANOVA model yielded significant main effects for time (F_2.11,67.38_ = 221.02, *p* < 0.001, η^2^_p_ = 0.87) and group (F_1,32_ = 6.01, *p* = 0.02, η^2^_p_ = 0.16). The group-by-time interaction effect was non-significant (F_2.11,67.38_ = 2.44, *p* = 0.09, η^2^_p_ = 0.07). Pairwise comparisons showed significantly higher V_T_ values at 20%, 40%, 60%, 80%, and 100% of each individual’s $\dot{V}{\text{O}}_{2\text{peak}}$ in the GWI+ group compared to the GWI- group (all p < 0.05; Table 5; Fig 1). Hedges’ *d* effect sizes for these comparisons ranged from 0.68 to 0.92.

**Table 5:** Comparison of mean (SD) ventilatory patterns between participants with positive and negative Gulf War Illness diagnoses.

	Full sample			Matched subgroup		
	Mean (SD)		Effect size (95% CI)	Mean (SD)		Effect size (95% CI)
	GWI + (n = 20)	GWI–(n = 14)		GWI + (n = 11)	GWI–(n = 11)	
${\dot{V}}_{\text{E}}$, **(l·min**^**-1**^ **)**						
**Rest**	17.02 (4.2)	16.23 (3.34)	0.2 (-0.48, 0.89)	15.01 (4.34)	16.60 (3.39)	-0.39 (-1.24, 0.45)
**20%**	22.22 (5.72)	20.77 (4.18)	0.27 (-0.41, 0.96)	19.61 (5.91)	21.45 (3.70)	-0.36 (-1.2, 0.48)
**40%**	29.65 (7.36)	28.66 (7.26)	0.13 (-0.55, 0.82)	26.44 (8.26)	29.73 (7.25)	-0.41 (-1.25, 0.44)
**60%**	39.2 (10.18)	38.29 (11.54)	0.08 (-0.6, 0.77)	34.20 (9.48)	40.09 (11.8)	-0.53 (-1.38, 0.32)
**80%**	51.91 (13.36)	50.21 (15.19)	0.12 (-0.57, 0.80)	47.82 (13.54)	52.79 (15.32)	-0.33 (-1.17, 0.51)
**100%**	68.89 (18.25)	63.48 (20.16)	0.28 (-0.41, 0.96)	63.95 (18.86)	66.76 (21.14)	-0.14 (-0.90, 0.70)
**V**_**T**_ **(liters)**						
**Rest**	0.95 (0.26)	0.78 (0.22)	0.68 (-0.02, 1.38)	0.88 (0.29)	0.81 (0.24)	0.25 (-0.59, 1.09)
**20%**	1.22 (0.33)	0.93 (0.26)	0.92 (0.21, 1.64)	1.13 (0.39)	0.96 (0.29)	0.48 (-0.37, 1.33)
**40%**	1.54 (0.39)	1.2 (0.41)	0.84 (0.13, 1.55)	1.44 (0.46)	1.25 (0.44)	0.41 (-0.44, 1.25)
**60%**	1.81 (0.45)	1.41 (0.51)	0.84 (0.13, 1.55)	1.72 (0.5)	1.46 (0.56)	0.47 (-0.37, 1.32)
**80%**	2.03 (0.47)	1.64 (0.52)	0.77 (0.07, 1.48)	1.96 (0.56)	1.71 (0.55)	0.44 (-0.41, 1.28)
**100%**	2.17 (0.49)	1.78 (0.54)	0.75 (0.04, 1.45)	2.07 (0.53)	1.85 (0.57)	0.39 (-0.46, 1.23)

#### Matched subgroup

The V_T_ ANOVA model yielded a significant main effect for time (F_1.82,36.34_ = 145.84, *p* < 0.001, η^2^_p_ = 0.88), but the group effect was no longer significant (F_1,20_ = 1.11, *p* = 0.31, η^2^_p_ = 0.05). The group-by-time interaction effect remained non-significant (F_1.82,36.34_ = 0.86, *p* = 0.42, η^2^_p_ = 0.04).

### Respiratory frequency

#### Full sample

The *f*_R_ ANOVA model yielded a significant main effect for time (F_2.46,78.78_ = 70.63, *p* < 0.001, η^2^_p_ = 0.69) and group (F_1,32_ = 9.87, *p* = 0.004, η^2^_p_ = 0.24). The group-by-time interaction effect was non-significant (F_2.46,78.78_ = 1.3, *p* = 0.28, η^2^_p_ = 0.04). Pairwise comparisons showed significantly lower *f*_R_ values at rest and 20%, 40%, 60%, and 80% of each individual’s $\dot{V}{\text{O}}_{2\text{peak}}$ in the GWI+ group compared to the GWI- group (all p < 0.05; Table 6; Fig 1). Hedges’ *d* effect sizes for these comparisons ranged from -0.61 to -1.05.

**Table 6:** Continued comparison of mean (SD) ventilatory patterns between participants with positive and negative Gulf War Illness diagnoses.

	Full study sample			Matched subgroup		
	Mean (SD)		Effect size (95% CI)	Mean (SD)		Effect size (95% CI)
	GWI + (n = 20)	GWI–(n = 14)		GWI + (n = 11)	GWI–(n = 11)	
***f***_**R**_ **(breath·min**^**-1**^ **)**						
**Rest**	19.6 (4.5)	22.48 (4.78)	-0.61 (-1.31, 0.09)	18.54 (3.35)	22.44 (4.77)	-0.91 (-1.79, -0.04)
**20%**	19.44 (4.31)	24.36 (4.96)	-1.05 (-1.77, -0.32)	18.62 (3.64)	24.67 (4.95)	-1.35 (-2.27, -0.42)
**40%**	20.29 (3.63)	26.05 (4.81)	-1.36 (-2.11, -0.6)	19.56 (3.27)	26.37 (5.37)	-1.48 (-2.42, -0.54)
**60%**	22.61 (5.12)	29.2 (7.38)	-1.05 (-1.78, -0.32)	20.83 (3.28)	29.78 (8.13)	-1.39 (-2.33, -0.46)
**80%**	26.31 (5.01)	31.83 (6.71)	-0.94 (-1.66, -0.22)	25.34 (4.27)	32.22 (7.36)	-1.10 (-2, -0.21)
**100%**	32.74 (7.78)	36.37 (6)	-0.50 (-1.19, 0.19)	31.95 (6.62)	36.81 (6.53)	-0.71 (-1.58, 0.15)

**Note**. *f*_R_ = respiratory frequency; **GWI+** = positive diagnosis for Gulf War Illness; **GWI-** = negative diagnosis for Gulf War Illness; ${\dot{\text{V}}}_{\text{E}}$ = minute ventilation; **V**_**T**_ = tidal volume. Mean values are expressed relative to each individual’s $\dot{V}{\text{O}}_{2\text{peak}}$ to facilitate between-group comparisons across similar intensities (i.e., rest, 20%, 40%, 60%, 80%, and 100% $\dot{V}{\text{O}}_{2}$ peak). Hedges’ *d* effect sizes were calculated by dividing the mean difference between GWI+ and GWI- by the pooled standard deviation. 95% Confidence intervals were calculated by adding or subtracting the product of the standard error of the effect size and 1.96 to or from each effect size.

#### Matched subgroup

**Fig 2:**
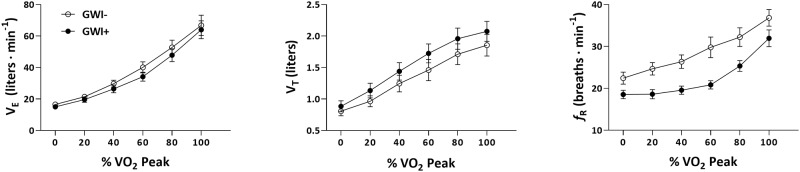
Time course of exercise ventilatory variables (${\dot{V}}_{\text{E}}$, V_T_, and *f*_R_) expressed as a function of relative intensity ($\dot{V}{\text{O}}_{2}$ peak) for the matched subgroup (GWI+ = 11, GWI- = 11). Cases (GWI+) and controls (GWI-) are represented in filled and open circles, respectively. Values are mean ± SE.

The *f*_R_ ANOVA model yielded a significant main effect for time (F_3.26,65.2_ = 54.01, *p* < 0.001, η^2^_p_ = 0.73) and group (F_1,20_ = 10.88, *p* = 0.004, η^2^_p_ = 0.35). The group-by-time interaction effect was non-significant (F_3.26,65.2_ = 1.54, *p* = 0.21, η^2^_p_ = 0.07). Pairwise comparisons showed significantly lower *f*_R_ values at rest, and 20%, 40%, 60%, and 80% of each individual’s $\dot{V}{\text{O}}_{2\text{peak}}$ in the GWI+ group compared to the GWI- group (all p < 0.05; Table 6; Fig 2). Hedges’ *d* effect sizes for these comparisons ranged from -0.91 to -1.48.

## Discussion

The present study investigated ventilatory patterns (${\dot{V}}_{\text{E}}$, V_T_, and *f*_R_) during maximal cardiopulmonary exercise (CPET) among veterans with GWI and controls. As expected, ${\dot{V}}_{\text{E}}$, V_T_, and *f*_R_ increased with increasing exercise intensity across groups (Fig 1). Veterans with GWI adopted a unique exercise ventilatory pattern; however, this pattern was contrary to our hypothesis and characterized by greater depth (V_T_) and reduced frequency (*f*_R_) of breathing relative to controls. According to the ANOVA models, the effect of group significantly accounted for 15 and 25% of the variability in V_T_ and *f*_R_, respectively. Moreover, *post-hoc* pairwise comparisons at each time-point during exercise (i.e. 20%, 40%, 60%, 80%, and 100% $\dot{V}{\text{O}}_{2}$ peak) revealed moderate-to-large between-group effects for V_T_ at all time-points during exercise (Hedges’ *d*_range_: 0.75–0.92). For *f*_R_, *post-hoc* pairwise comparisons revealed large and significant group effects for 20%, 40%, 60%, 80% of $\dot{V}{\text{O}}_{2}$ peak (Hedges’ *d*_range_: 0.94–1.36), but not 100% of $\dot{V}{\text{O}}_{2}$ peak. After performing a sub-group analysis of participants who were closely matched for major determinants of vital capacity (i.e., race, sex, age, height), the group effect remained significant for *f*_R_ but not for V_T_ (Fig 2). This suggests that *f*_R_ is an especially robust aspect of ${\dot{V}}_{\text{E}}$ distinguishing Veterans with and without GWI. This disparate breathing strategy was observed despite non-significant between-group differences for ${\dot{V}}_{\text{E}}$ (full sample and matched subgroup), which is important to note given that the components of ${\dot{V}}_{\text{E}}$ (V_T_ and *f*_R_) are frequently overlooked [1] despite their clinical relevance (e.g., tachypnea in congestive heart failure).

To our knowledge, of the 10 CPET studies involving Gulf War veterans [12–21], only one study has reported detailed cardiopulmonary function of Gulf War veterans as assessed during a maximal CPET [12, 13]. In this study, CPET performance was found to be similar between veterans with and without GWI at peak and submaximal intensities, apart from peak ${\dot{V}}_{\text{E}}$ which was reduced in GWI. This effect appears to be driven by a reduced *f*_R_ (*g* = -0.46) among ill Gulf War veterans in comparison to healthy Gulf War veterans [13], similar to that observed in the present study. Maximal exercise ventilatory responses during maximal testing from related populations (e.g., chronic fatigue syndrome [CFS] and fibromyalgia) are limited, but have generally found similar *f*_R_ and V_T_ between cases and controls in patients with CFS [30, 31], CFS and fibromyalgia [30], or fibromyalgia alone [32]. It is important to note that in these studies of patients with CFS and/or fibromyalgia, a majority of the sample was female which may not provide an appropriate comparison to the participants of the present study.

Published reference data for *f*_R_ and V_T_ are scant for cycling exercise but are available for maximal treadmill testing [33]. Differences in operating lung volumes between cycling and treadmill exercise, most notably larger V_T_ during cycling [34], precludes the application of treadmill based prediction equations for interpreting cycling studies. Neder et al. reported average peak *f*_R_ obtained during incremental cycle exercise stratified by age ranges (20–39, 40–59, and 60–80 years) and sex at four different peak ${\dot{V}}_{\text{E}}$ levels (20, 40, 60, and 80 L∙min^-1^) from 120 sedentary adults (c.f. Table 2 [35]). Using these data, we estimated a predicted *f*_R_ value for each participant based on age range, sex and approximate peak ${\dot{V}}_{\text{E}}$. Predicted *f*_R_ was 99.4±22.4% and 116.4±23.5% for cases (GWI+) and controls (GWI-), respectively. Although we would have expected predicted *f*_R_ to be even lower among veterans with GWI, there are several important factors that may account for these observed differences including the lack of precision around age ranges and peak ${\dot{V}}_{\text{E}}$ levels, race and ethnicity of the reference sample, and instrumentation. Regarding the latter, Neder et al. [35] utilized a nose clip and mouthpiece to acquire ventilatory variables which is known to result in reduced *f*_R_ and increased V_T_ in comparison to an oronasal mask [36], which was used in the present study.

At exercise onset and during lower intensity workloads, increases in ${\dot{V}}_{\text{E}}$ are primarily driven by V_T_ whereby further increases in ${\dot{V}}_{\text{E}}$ at greater workloads and at peak exercise are achieved primarily through increased *f*_R_. This typical ventilatory response to exercise is well characterized and thought to reflect a strategy to maximize metabolic efficiency [37]. Excessive increases in either V_T_ or *f*_R_ deviate from this principle and result in increased work of breathing and increased physiologic dead space. Accurate measurement of work of breathing and dead space ventilation (V_D_/V_T_) require invasive procedures (i.e., esophageal pressures and arterial catheter) that were not employed in the present study; therefore, we cannot definitively rule out their contribution. However, abnormal findings such as increased V_D_/V_T_ and work of breathing appear unlikely for several reasons. First, we computed non-invasive estimates of V_D_/V_T_ [38] and work of breathing [39] and found similar results among our cases and controls that were within normal limits. Increased V_D_/V_T_ and work of breathing are most common among individuals with chronic lung disease, but lung disease was exclusionary for this study. Further, we observed no evidence of ventilatory limitation at rest via spirometry (Table 2) nor during peak exercise (VE/MVV ≤ 80%; Table 4). That V_T_ continued to increase throughout exercise also supports the lack of a ventilatory limitation, or more specifically the absence of a restrictive process that would limit V_T_ expansion. Lastly, both groups demonstrated a similar ratio of V_T_ to inspiratory capacity (V_T_:IC) throughout exercise which was within normal limits (V_T_:IC ≤ 0.8; Table 4). We conclude that there is little evidence to support an underlying subclinical pulmonary pathology among cases with GWI+ that may account for the reduced rate and greater depth of breathing observed in the present study. Moreover, our matched subgroup analysis further confirms the robustness of these findings. We posit that the observed ventilatory pattern might instead reflect a learned strategy to mitigate exercise-induced pain and/or unique metabolic demands associated with GWI.

As reviewed by Forster et al. [40], numerous experimental studies support a neural feed-forward (i.e., central command) mediation of exercise hyperpnoea, suggesting central nervous system control of breathing involves a behavioral or learned mechanism. Long-term modulation of the exercise ventilatory response has been demonstrated experimentally in both humans [41, 42] and goats [43] via a conditioning paradigm whereby exercise is paired with added external dead space in order to induce hypercapnia. Following 2–8 visits of conditioning and removal of added dead space, subsequent steady-state normocapnic exercise resulted in increased V_T_ during early exercise. In a follow-up study to determine whether long-term modulation was driven primarily by hypercapnia or increased V_T_, Turner and Stewart [44] employed inspiratory resistive loading as a model of associative conditioning to avoid inducing hypercapnia, and observed long-term modulation of the early exercise ventilatory response of similar magnitude and duration to the aforementioned prior work. Data from these studies suggest that at least some component of the normal exercise ventilatory response may be learned, and increased V_T_, rather than hypercapnia, may provide the requisite stimulus. The persistence of these adaptations, however, appears temporary which likely reflects the limited exposure to conditioning. Wood et al. [42] support this conclusion and suggest that a permanent breathing pattern adaptation is unlikely to occur following only an acute exposure. Whether alterations to the exercise ventilatory response (i.e., increased V_T_) can be sustained in humans with chronic conditioning remains unclear.

Cook et al.[16] have previously found that Gulf War veterans with chronic pain perceive exercise as more painful and effortful than healthy veterans without pain, and also have greater thermal pain sensitivity post-exercise. Based on these results, we initially hypothesized that *f*_R_ would be exaggerated in GWI given that non-metabolic stimuli, such as pain, are well known to elicit robust increases in *f*_R_ in healthy adults [1]. However, perhaps the ventilatory response to pain is unique among those with chronic illness who have been managing chronic pain symptoms for many years? In support, fear of exacerbating pain symptoms secondary to exercise appears to be prevalent in civilians with medically unexplained widespread chronic musculoskeletal pain [45] and this perception may lead to a conscious or subconscious alteration in exercise ventilatory patterns (i.e., slower and deeper). There are now several studies that support the notion that pain is influenced by respiration whereby deep and slow breathing reduces pain intensity and perception [46–49]. Whether veterans with GWI adopt a deep and slow ventilatory pattern to mitigate exercise-induced pain cannot be determined from our data, but warrants future investigation.

Notwithstanding the limitations of a cross-sectional study, there are additional aspects of this study that should be considered when interpreting these findings. First, although the size of the participant sample is within the range of previous CPET studies involving Gulf War veterans, GWI is a heterogeneous chronic multisymptom illness and replication of this work with a larger sample size is necessary to enhance generalizability. As previously mentioned, the paucity of published cardiopulmonary data from exercise studies in GWI precludes a thorough comparison with work from other investigators. In light of the present findings, future studies that include a more sophisticated analysis of ventilatory patterns during exercise to determine operating lung volumes (e.g., serial inspiratory capacity maneuvers) as well as measurement of the work of breathing via esophageal pressures appear warranted to fully appreciate the mechanical and neural factors that contribute to this presentation of increased depth of breathing during exercise. Finally, we did not comprehensively assess symptomatic changes during exercise. Considering the frequent use of exercise to elucidate illness pathophysiology and that the gold standard for GWI case assignment relies on self-report methods, an intuitive step when measuring physiological responses to exercise is to also measure perceptual responses. Thus, incorporating subjective measures for symptoms that are characteristic of the illness (e.g., pain, fatigue) and are sensitive to acute exercise-induced changes may aid future attempts to link changes in physiological parameters to pathophysiological indices of chronic multisymptom illnesses such as GWI [50].

### Conclusions

Excessive increase in either V_T_ or *f*_R_ in response to exercise is an inefficient ventilatory strategy that contributes to poor performance and exertional symptoms; however, V_T_ and *f*_R_ are infrequently reported and often overlooked. In the present study, we observed a unique exercise ventilatory pattern among veterans with GWI characterized predominantly by an attenuated *f*_R_ response to maximal exercise. The present study was not designed to assess the mechanism(s) of this response, but warrants further investigation. Confirmation of these findings may also provide a rationale on which to guide exercise prescription for veterans with GWI (e.g., wearable devices for *f*_R_ [51]). In conclusion, researchers and clinicians should consider looking beyond ${\dot{V}}_{\text{E}}$ and separately consider V_T_ and *f*_R_ responses to exercise, which appear to offer unique insight with respect to exercise hyperpnoea.

## Supporting information

S1 Table Matched-pair demographics. For each case (GWI+) and control (GWI-) pair, we report i) race and ethnicity, ii) birth sex, iii) age in years, iv) height in centimeters, and v) forced vital capacity (FVC). Absolute differences for each variable for each pair (Δ = case—control) are reported for age, height and FVC. Overall, we were able to match evenly by sex and race/ethnicity. Other factors were very similar as indicated by the low average differences observed for age (3.7 years), height (6.5 cm), and FVC (0.2 L). Note that acceptable and repeatable spirometry were not available for one Veteran (pair 8).
(DOCX)

## References

[1] Tipton MJ, Harper A, Paton JF, Costello JT. The human ventilatory response to stress: rate or depth? The Journal of Physiology. 2017. 10.1113/JP274596 PMC5577533 28650070

[2] Nicolo A, Marcora SM, Bazzucchi I, Sacchetti M. Differential control of respiratory frequency and tidal volume during high-intensity interval training. Exp Physiol. 2017;102(8):934–49. 10.1113/EP086352 . 28560751

[3] Nicolo A, Bazzucchi I, Haxhi J, Felici F, Sacchetti M. Comparing continuous and intermittent exercise: an "isoeffort" and "isotime" approach. PLoS One. 2014;9(4):e94990 10.1371/journal.pone.0094990 . 24736313 PMC3988078

[4] Nicolo A, Marcora SM, Sacchetti M. Respiratory frequency is strongly associated with perceived exertion during time trials of different duration. J Sports Sci. 2016;34(13):1199–206. 10.1080/02640414.2015.1102315 . 26503587

[5] Bell HJ, Duffin J. Rapid increases in ventilation accompany the transition from passive to active movement. Respir Physiol Neurobiol. 2006;152(2):128–42. Epub 2005/09/13. 10.1016/j.resp.2005.07.008 . 16153897

[6] Thornton JM, Guz A, Murphy K, Griffith AR, Pedersen DL, Kardos A, et al Identification of higher brain centres that may encode the cardiorespiratory response to exercise in humans. J Physiol. 2001;533(Pt 3):823–36. 10.1111/j.1469-7793.2001.00823.x . 11410638 PMC2278657

[7] ATS/ACCP Statement on Cardiopulmonary Exercise Testing. 2003;167(2):211–77. 10.1164/rccm.167.2.211 . 12524257

[8] White RF, Steele L, O'Callaghan JP, Sullivan K, Binns JH, Golomb BA, et al Recent research on Gulf War illness and other health problems in veterans of the 1991 Gulf War: Effects of toxicant exposures during deployment. Cortex. 2016;74:449–75. 10.1016/j.cortex.2015.08.022 26493934 PMC4724528

[9] Kelsall HL, Sim MR, Forbes AB, McKenzie DP, Glass DC, Ikin JF, et al Respiratory health status of Australian veterans of the 1991 Gulf War and the effects of exposure to oil fire smoke and dust storms. Thorax. 2004;59(10):897–903. 10.1136/thx.2003.017103 15454658 PMC1746848

[10] Karlinsky JB, Blanchard M, Alpern R, Eisen SA, Kang H, Murphy FM, et al Late prevalence of respiratory symptoms and pulmonary function abnormalities in Gulf War I Veterans. Arch Intern Med. 2004;164(22):2488–91. 10.1001/archinte.164.22.2488 15596641

[11] Medinger AE, Chan TW, Arabian A, Rohatgi PK. Interpretive algorithms for the symptom-limited exercise test: assessing dyspnea in Persian Gulf war veterans. Chest. 1998;113(3):612–8. 10.1378/chest.113.3.612 9515833

[12] Nagelkirk P, Cook D, Peckerman A, Kesil W, Sakowski T, Natelson B, et al Aerobic capacity of Gulf War veterans with chronic fatigue syndrome. Military medicine. 2003;168(9):750 14529252

[13] Cook DB, Nagelkirk PR, Peckerman A, Poluri A, LaManca JJ, Natelson BH. Perceived exertion in fatiguing illness: Gulf War veterans with chronic fatigue syndrome. Medicine and science in sports and exercise. 2003;35(4):569–74. 10.1249/01.MSS.0000058438.25278.33 12673138

[14] Broderick G, Ben-Hamo R, Vashishtha S, Efroni S, Nathanson L, Barnes Z, et al Altered immune pathway activity under exercise challenge in Gulf War Illness: An exploratory analysis. Brain, Behavior, and Immunity. 2013;28:159–69. 10.1016/j.bbi.2012.11.007. 10.1016/j.bbi.2012.11.007 23201588

[15] Broderick G, Kreitz A, Fuite J, Fletcher MA, Vernon SD, Klimas N. A pilot study of immune network remodeling under challenge in Gulf War Illness. Brain, Behavior, and Immunity. 2011;25(2):302–13. 10.1016/j.bbi.2010.10.011 20955779

[16] Cook DB, Stegner AJ, Ellingson LD. Exercise Alters Pain Sensitivity in Gulf War Veterans With Chronic Musculoskeletal Pain. The Journal of Pain. 2010;11(8):764–72. 10.1016/j.jpain.2009.11.010 20338824

[17] Donta ST C DJ EJ CC, et al Cognitive behavioral therapy and aerobic exercise for gulf war veterans' illnesses: A randomized controlled trial. JAMA. 2003;289(11):1396–404. 10.1001/jama.289.11.1396 12636462

[18] Rayhan RU, Raksit MP, Timbol CR, Adewuyi O, VanMeter JW, Baraniuk JN. Prefrontal lactate predicts exercise-induced cognitive dysfunction in Gulf War Illness. American Journal of Translational Research. 2013;5(2):212–23. 23573365 PMC3612516

[19] Rayhan RU, Stevens BW, Raksit MP, Ripple JA, Timbol CR, Adewuyi O, et al Exercise Challenge in Gulf War Illness Reveals Two Subgroups with Altered Brain Structure and Function. PLoS ONE. 2013;8(6):e63903 10.1371/journal.pone.0063903 23798990 PMC3683000

[20] Smylie AL, Broderick G, Fernandes H, Razdan S, Barnes Z, Collado F, et al A comparison of sex-specific immune signatures in Gulf War illness and chronic fatigue syndrome. BMC Immunology. 2013;14(1):29 10.1186/1471-2172-14-29 23800166 PMC3698072

[21] Whistler T, Fletcher M, Lonergan W, Zeng XR, Lin JM, LaPerriere A, et al Impaired immune function in Gulf War Illness. BMC Medical Genomics. 2009;2(1):12. 19265525 10.1186/1755-8794-2-12 PMC2657162

[22] IOM. Chronic Multisymptom Illness in Gulf War Veterans: Case Definitions Reexamined. Washington, DC: The National Academies Press; 2014 130 p. 25590117

[23] Steele L. Prevalence and patterns of Gulf War illness in Kansas veterans: association of symptoms with characteristics of person, place, and time of military service. Am J Epidemiol. 2000;152(10):992–1002. Epub 2000/11/25. 10.1093/aje/152.10.992 . 11092441

[24] Krupp LB, LaRocca NG, Muir-Nash J, Steinberg AD. The fatigue severity scale. Application to patients with multiple sclerosis and systemic lupus erythematosus. Arch Neurol. 1989;46(10):1121–3. 10.1001/archneur.1989.00520460115022 . 2803071

[25] Falvo MJ, Serrador JM, McAndrew LM, Chandler HK, Lu SE, Quigley KS. A retrospective cohort study of U.S. service members returning from Afghanistan and Iraq: is physical health worsening over time? BMC Public Health. 2012;12:1124 Epub 2013/01/01. 10.1186/1471-2458-12-1124 . 23272950 PMC3543837

[26] Miller MR, Hankinson J, Brusasco V, Burgos F, Casaburi R, Coates A, et al Standardisation of spirometry. EurRespirJ. 2005;26(2):319–38. 10.1183/09031936.05.00034805 16055882

[27] Hankinson J, Odencrantz J, Fedan K. Spirometric Reference Values from a Sample of the General U.S. Population. American Journal of Respiratory and Critical Care Medicine. 1999;159(1):179–87. 10.1164/ajrccm.159.1.9712108 9872837

[28] Beaver WL, Wasserman K, Whipp BJ. A new method for detecting anaerobic threshold by gas exchange. J Appl Physiol (1985). 1986;60(6):2020–7. 10.1152/jappl.1986.60.6.2020 . 3087938

[29] Fritz CO, Morris PE, Richler JJ. Effect size estimates: current use, calculations, and interpretation. J Exp Psychol Gen. 2012;141(1):2–18. Epub 2011/08/10. 10.1037/a0024338 . 21823805

[30] Cook DB, Nagelkirk PR, Poluri A, Mores J, Natelson BH. The influence of aerobic fitness and fibromyalgia on cardiorespiratory and perceptual responses to exercise in patients with chronic fatigue syndrome. Arthritis Rheum. 2006;54(10):3351–62. 10.1002/art.22124 17009309

[31] Inbar O, Dlin R, Rotstein A, Whipp BJ. Physiological responses to incremental exercise in patients with chronic fatigue syndrome. Med Sci Sports Exerc. 2001;33(9):1463–70. Epub 2001/08/31. 10.1097/00005768-200109000-00007 . 11528333

[32] Sanudo B, Galiano D. Using cardiovascular parameters and symptom severity to prescribe physical activity in women with fibromyalgia. Clin Exp Rheumatol. 2009;27(5 Suppl 56):S62–6. Epub 2010/03/12. . 20074442

[33] Loe H, Steinshamn S, Wisloff U. Cardio-respiratory reference data in 4631 healthy men and women 20–90 years: the HUNT 3 fitness study. PLoS One. 2014;9(11):e113884 Epub 2014/11/27. 10.1371/journal.pone.0113884 . 25426954 PMC4245230

[34] Tanner DA, Duke JW, Stager JM. Ventilatory patterns differ between maximal running and cycling. Respir Physiol Neurobiol. 2014;191:9–16. Epub 2013/11/12. 10.1016/j.resp.2013.10.011 . 24211317

[35] Neder JA, Dal Corso S, Malaguti C, Reis S, De Fuccio MB, Schmidt H, et al The pattern and timing of breathing during incremental exercise: a normative study. Eur Respir J. 2003;21(3):530–8. Epub 2003/03/29. 10.1183/09031936.03.00045402 . 12662013

[36] Hirsch JA, Bishop B. Human breathing patterns on mouthpiece or face mask during air, CO2, or low O2. J Appl Physiol Respir Environ Exerc Physiol. 1982;53(5):1281–90. Epub 1982/11/01. 10.1152/jappl.1982.53.5.1281 . 6816769

[37] Sheel AW, Romer LM. Ventilation and respiratory mechanics. Compr Physiol. 2012;2(2):1093–142. 10.1002/cphy.c100046 . 23798297

[38] Pianosi P, D'Souza SJ, Esseltine DW, Charge TD, Coates AL. Ventilation and gas exchange during exercise in sickle cell anemia. Am Rev Respir Dis. 1991;143(2):226–30. Epub 1991/02/01. 10.1164/ajrccm/143.2.226 . 1990932

[39] Aaron EA, Johnson BD, Seow CK, Dempsey JA. Oxygen cost of exercise hyperpnea: measurement. J Appl Physiol (1985). 1992;72(5):1810–7. Epub 1992/05/01. 10.1152/jappl.1992.72.5.1810 . 1601790

[40] Forster HV, Haouzi P, Dempsey JA. Control of Breathing During Exercise. Comprehensive Physiology: John Wiley & Sons, Inc.; 2011. 10.1002/cphy.c100045 23728984

[41] Turner DL, Sumners DP. Associative conditioning of the exercise ventilatory response in humans. Respir Physiol Neurobiol. 2002;132(2):159–68. Epub 2002/08/06. . 12161329 10.1016/s1569-9048(02)00075-7

[42] Wood HE, Fatemian M, Robbins PA. A learned component of the ventilatory response to exercise in man. J Physiol. 2003;553(Pt 3):967–74. 10.1113/jphysiol.2003.047597 . 14514870 PMC2343621

[43] Martin PA, Mitchell GS. Long-term modulation of the exercise ventilatory response in goats. J Physiol. 1993;470:601–17. 10.1113/jphysiol.1993.sp019877 . 8308746 PMC1143936

[44] Turner D, Stewart JD. Associative conditioning with leg cycling and inspiratory resistance enhances the early exercise ventilatory response in humans. Eur J Appl Physiol. 2004;93(3):333–9. 10.1007/s00421-004-1194-2 . 15375661

[45] Turk DC, Robinson JP, Burwinkle T. Prevalence of fear of pain and activity in patients with fibromyalgia syndrome. The Journal of Pain. 2004;5(9):483–90. 10.1016/j.jpain.2004.08.002 15556826

[46] Zautra AJ, Fasman R, Davis MC, Craig AD. The effects of slow breathing on affective responses to pain stimuli: an experimental study. Pain. 2010;149(1):12–8. Epub 2010/01/19. 10.1016/j.pain.2009.10.001 . 20079569

[47] Chalaye P, Goffaux P, Lafrenaye S, Marchand S. Respiratory effects on experimental heat pain and cardiac activity. Pain Med. 2009;10(8):1334–40. Epub 2009/08/13. 10.1111/j.1526-4637.2009.00681.x . 19671085

[48] Busch V, Magerl W, Kern U, Haas J, Hajak G, Eichhammer P. The effect of deep and slow breathing on pain perception, autonomic activity, and mood processing—an experimental study. Pain Med. 2012;13(2):215–28. Epub 2011/09/24. 10.1111/j.1526-4637.2011.01243.x . 21939499

[49] Grant JA, Rainville P. Pain sensitivity and analgesic effects of mindful states in Zen meditators: a cross-sectional study. Psychosom Med. 2009;71(1):106–14. Epub 2008/12/17. 10.1097/PSY.0b013e31818f52ee . 19073756

[50] Lindheimer JB, Meyer JD, Stegner AJ, Dougherty RJ, Van Riper SM, Shields M, et al Symptom variability following acute exercise in myalgic encephalomyelitis/chronic fatigue syndrome: a perspective on measuring post-exertion malaise. Fatigue: Biomedicine, Health & Behavior. 2017;5(2):69–88. 10.1080/21641846.2017.1321166

[51] Nicolo A, Massaroni C, Passfield L. Respiratory Frequency during Exercise: The Neglected Physiological Measure. Front Physiol. 2017;8:922 Epub 2018/01/13. 10.3389/fphys.2017.00922 . 29321742 PMC5732209

