# Supplementary material for: Veterans with Gulf War Illness exhibit distinct respiratory patterns during maximal cardiopulmonary exercise
Source: PLoS One. 2019 Nov 12;14(11):e0224833. doi: 10.1371/journal.pone.0224833 (PMC6850551; doi:10.1371/journal.pone.0224833)
Supplement: S1 Table — For each case (GWI+) and control (GWI-) pair, we report i) race and ethnicity, ii) birth sex, iii) age in years, iv) height in centimeters, and v) forced vital capacity (FVC). Absolute differences for each variable for each pair (Δ = case—control) are reported for age, height and FVC. Overall, we were able to match evenly by sex and race/ethnicity. Other factors were very similar as indicated by the low average differences observed for age (3.7 years), height (6.5 cm), and FVC (0.2 L). Note that acceptable and repeatable spirometry were not available for one Veteran (pair 8). (DOCX) [file pone.0224833.s001.docx]

**Supporting Information**

| **Pair** | **Group** | **Race/Ethnicity** | **Birth Sex** | **Age (yrs)** | **Age Δ** | **Ht (cm)** | **Ht Δ** | **FVC (L)** | **FVC Δ** |
| --- | --- | --- | --- | --- | --- | --- | --- | --- | --- |
| 1 | GWI- | White/Non-Hispanic | F | 60 | 2 | 157 | 2 | 3.65 | 0.05 |
| 1 | GWI+ | White/Non-Hispanic | F | 58 |  | 155 |  | 3.6 |  |
| 2 | GWI- | Black/Non-Hispanic | F | 68 | 13 | 165 | 15 | 2.24 | 0.25 |
| 2 | GWI+ | Black/Non-Hispanic | F | 55 |  | 150 |  | 2.49 |  |
| 3 | GWI- | Black/Non-Hispanic | F | 56 | 5 | 163 | 2 | 3.14 | 0.23 |
| 3 | GWI+ | Black/Non-Hispanic | F | 51 |  | 161 |  | 3.37 |  |
| 4 | GWI- | American Indian/Alaskan Native | M | 47 | 2 | 176 | 7 | 4.58 | 0.27 |
| 4 | GWI+ | American Indian/Alaskan Native | M | 45 |  | 183 |  | 4.85 |  |
| 5 | GWI- | Black African American/Non-Hispanic | M | 49 | 3 | 177 | 11 | 4.92 | 0.01 |
| 5 | GWI+ | Black African American/Non-Hispanic | M | 52 |  | 188 |  | 4.93 |  |
| 6 | GWI- | Black African American/Non-Hispanic | M | 49 | 4 | 174 | 2 | 4.07 | 0.05 |
| 6 | GWI+ | Black African American/Non-Hispanic | M | 53 |  | 176 |  | 4.12 |  |
| 7 | GWI- | Black African American/Non-Hispanic | M | 48 | 6 | 168 | 7 | 3.56 | 0.37 |
| 7 | GWI+ | Black African American/Non-Hispanic | M | 42 |  | 175 |  | 3.93 |  |
| 8 | GWI- | Black African American/Non-Hispanic | M | 47 | 1 | 187 | 9 | 4.75 | - |
| 8 | GWI+ | Black African American/Non-Hispanic | M | 46 |  | 178 |  | - |  |
| 9 | GWI- | White/Non-Hispanic | M | 47 | 2 | 175 | 2 | 4.35 | 0.37 |
| 9 | GWI+ | White/Non-Hispanic | M | 49 |  | 173 |  | 4.72 |  |
| 10 | GWI- | White/Non-Hispanic | M | 63 | 1 | 168 | 2 | 4.07 | 0.15 |
| 10 | GWI+ | White/Non-Hispanic | M | 62 |  | 170 |  | 4.22 |  |
| 11 | GWI- | White/Non-Hispanic | M | 58 | 2 | 163 | 12 | 4.17 | 0.24 |
| 11 | GWI+ | White/Non-Hispanic | M | 56 |  | 175 |  | 4.41 |  |

**Supplemental Table A – Matched-Pair Demographics**

For each case (GWI+) and control (GWI-) pair, we report i) race and ethnicity, ii) birth sex, iii) age in years, iv) height in centimeters, and v) forced vital capacity (FVC). Absolute differences for each variable for each pair (Δ = case – control) are reported for age, height and FVC. Overall, we were able to match evenly by sex and race/ethnicity. Other factors were very similar as indicated by the low average differences observed for age (3.7 years), height (6.5 cm), and FVC (0.2 L). Note that acceptable and repeatable spirometry were not available for one Veteran (pair 8).
